# Too serious to ignore: The epidemiologic and economic burden of home injuries in the Southwest Region of Cameroon—A community-based study

**DOI:** 10.1371/journal.pone.0274686

**Published:** 2022-09-22

**Authors:** Eunice Oben Bessem Cole, S. Ariane Christie, Rasheedat Oke, Girish Motwani, Drusia Dickson, William Chendjou, Mbiarikai Mbianyor, Rochelle Dicker, Catherine Juillard, Alain Chichom-Mefire

**Affiliations:** 1 Department of Surgery, Faculty of Health Sciences, University of Buea, Buea, Cameroon; 2 Department of Surgery, Center for Global Surgical Studies, University of California, San Francisco, San Francisco, California, United States of America; 3 Department of Surgery, Program for the Advancement of Surgical Equity, University of California, Los Angeles, Los Angeles, California, United States of America; University Lyon 1 Faculty of Dental Medicine, FRANCE

## Abstract

**Background:**

Home injuries are an important cause of morbidity and mortality in high-income countries. In Sub-Saharan Africa, including Cameroon, many people live in unplanned settlements with poorly constructed houses, predisposing them to home injuries. However, little is known about the epidemiology and care-seeking behaviors of the domestically injured. In this study, our objective was to determine the epidemiology and care-seeking behaviors of home injuries in the Southwest Region of Cameroon.

**Methods:**

A sub-analyses of a larger descriptive cross-sectional community-based study on injury epidemiology in the preceding 12 months was conducted. Sampling was done using three-stage cluster sampling technique. Differences between groups were evaluated using Chi-squared and Adjusted Wald tests.

**Results:**

Of 8065 participants, 157 suffered home injuries giving an incidence of 19.6 (16.8–23.0 95% CI) cases per 1000-person years. Home injuries comprised 31.2% of all 503 injuries and affected more females (60.8%) and younger individuals (mean age (SE) 25.1 years (2.0)) than non-home injuries. The most common activity and mechanism of home injury was leisure/play (51%) and falls (37.9%) respectively. Amongst those with home injuries, 37.6% did not seek care from any care provider (versus 25.0% of non-home injuries, p = 0.004) and were more likely to seek treatment within the family or at home (p = 0.008) or at church (p = 0.010). Those with home injuries experienced a median of 14 disability days and 22.9% of families faced difficulties affording basic expenses (p = 0.001).

**Conclusion:**

Home injuries comprise about a third of the Southwest Region of Cameroon’s burden of injury and likely have a profound socioeconomic impact. Though these injuries cause severe disabilities, a large proportion of victims do not seek care from providers. Prevention efforts should address the design of homes and victims of home injury should be encouraged to utilize formal care services.

## Introduction

Injury is one of the leading causes of death and disability worldwide, with more than 90% of global deaths from injury occurring in low- and middle-income countries (LMICs) [1]. In 2012, about 10% of the mortality burden in Sub-Saharan Africa (SSA) was attributable to injuries [2]. The Global Burden of Disease study also found that unintentional injuries comprised the eighth leading cause of death in both SSA and Cameroon in 2016, resulting in estimated mortality rates of 26 per 100,000 population and 32 per 100,000 population, respectively [3].

Home injuries, defined as injuries occurring within the home and its premises, are a serious public health problem and an important cause of morbidity and mortality. Several studies carried out in the United States and Europe report a high incidence of home-related injury consultations, hospitalizations, and death [4–10]. In Europe every year, an estimated 32 million require admission and almost 110,000 die due to home injury [5]. In New Zealand alone, one study estimated that unintentional home injuries impose an annual social cost of about USD 9 billion annually [11, 12].

Some people in SSA, including in countries like Cameroon, are known to live in unplanned settlements with poorly constructed houses, further predisposing them to home injuries which have the potential to result in significant disability, loss of productivity, and death [11, 13–17]. Research from 1998 in South Africa demonstrated that more than three-quarters of all injuries were unintentional, most of which were sustained at home [18]. Despite the suspected high incidence, associated disability, and economic consequences, very little additional research has been carried out on home injuries in SSA, and to the best of our knowledge, none in Cameroon. Accurate information regarding the epidemiology of home injuries is critical to informing prevention strategies tailored to this specific health issue.

In a prior hospital-based study in Cameroon, in-home injuries comprised 20% of all injuries, second only to roads in terms of injury location [19]. Research carried out in several LMICs suggests that there is a low rate of utilization of formal care services [20–23]. Hospital-based data therefore may not accurately reflect the underlying burden of injury in the population, both underestimating the magnitude and introducing selection bias regarding the types of injuries reported. Additionally, little is known regarding the reasons injured individuals may or may not choose to seek formal care, further limiting our interpretation of hospital data.

To estimate the magnitude and burden of home injury, we conducted as part of a larger community-based study on injury in the Southwest Region of Cameroon, a sub-analysis to determine the epidemiology and outcomes of home injuries. We also sought to describe therapeutic itineraries of individuals injured in the home and assess factors influencing care-seeking decisions after sustaining a home injury. Additionally, we compared the epidemiology and care-seeking behavior of individuals injured at home to those injured in other locations and individuals with no injuries.

## Methods

### Study design, setting and population

This study was carried out as a sub-analysis of a cross-sectional community-based study between January and March 2017 in the Southwest Region of Cameroon, which has both urban and rural communities and is primarily Anglophone. The community-based study was powered to identify the incidence of injury that had occurred in the preceding one-year period and was carried out on a representative population, including people of all age groups and sexes. The one-year recall period was selected based on Mock et al.’s work, in order to prioritize information on the more severe, but less frequent injuries [24].

The study population consisted of all individuals residing in the SWR. Eligible households were those who consented to participate and had been living, eating, and sleeping for at least 6 months. Households without a member older than 18 years present after two attempts, those who did not understand the consent, or individuals not permanently residing in the SWR were excluded from the study.

### Sampling strategy

Study participants were selected using a three-staged cluster sampling framework. Details about the sampling strategy and sample size calculation are described elsewhere [25]. Briefly, the first and second sampling levels were the health districts and health areas, respectively, which were selected with probability of selection proportionate to population size. For the third level (households) a location point was randomly determined in the selected community and household sampling began with the settlement nearest to the starting point, then continued until the target value was reached (200 households per health area). The targeted sample size for the community-based survey was calculated using the World Health Organization’s (WHO) guidelines for community-based cluster surveys [25, 26]. A minimum sample size of 4,680 individuals was calculated for the larger study which was deliberately exceeded during data collection by approximately 50% at each site to account for multiple sub-analyses of relatively rare events.

### Survey administration and data collection

Nine Cameroonian students served as trained research assistants and administered a standard oral informed consent script and a pre-tested structured questionnaire (S1 Appendix) adapted for Cameroon from the WHO’s *Guidelines for conducting community surveys on injuries and violence* to an adult family representative selected from each household [26]. Basic information was collected from the representative on demographics of all family members, and on injury mechanism, outcome, and care-seeking behavior for family members who sustained home injuries within the study period. To maximize participation with survey results, participants were advised that they could abstain from providing an answer for any question which was important given the inclusion of questions on culturally uncomfortable subjects like payment remuneration and substance abuse. Participants did not receive any renumeration for their participation in the study. For the purposes of the survey, “injury” included any sudden bodily insult directly resulting in death or loss of routine daily activity by any family member for at least one day or bodily insult requiring treatment [27]. Home injury was defined as injury occurring at home or its immediate premises. Further details about questionnaire administration have been previously described [25].

### Data management and analysis

All data were periodically entered into University of California, San Francisco REDCap database [28]. The data was adjusted for clustering methodology using *svy* commands as appropriate and performed all analyses on Stata Version 14 [29]. Descriptive analyses were performed using frequencies, proportions, mean and standard errors (SEs) for continuous, normally distributed variables and medians with interquartile ranges (IQR) for nonparametric variables. Differences between groups were evaluated using Pearson’s Chi-squared and Fisher’s exact tests (as applicable) for categorical variables and Adjusted Wald tests for quantitative variables. Statistical significance was set at p ≤0.05. Data missingness varied by question but remained less than 10% for all questions in the sample. We excluded for this sub-analysis those without injury location which is the main variable for this sub-study.

### Ethical considerations

Ethical approval including the consent procedure for participants was obtained from the Institutional Review Boards of the University of California, San Francisco (IRB# 15–18424) and the University of Douala (No IEC-UD/694/10/2016/A). A family representative selected from each household was administered a standard oral informed consent script.

## Results

A total of 1,551 households were approached for consent with 151 (9.7%) found to be ineligible and 113 (7.3%) households refused consent. Thus, 8065 participants were included in the community-based study from 1,287 households that provided consent. Overall, 503 injuries were reported, of which 157 (31.2% of all injuries) were home injuries, yielding an incidence of 19.6 home injury cases per 1000 person-years (95% CI 16.8–23.0 cases per 1000-person years). A total of 10 injuries did not report on location of injury and were excluded from analysis.

### Socio-demographic characteristics

The majority of home injury victims were significantly females (60.8% p = 0.003) and lived in rural settings (58.4%) (Table 1). The mean age (SE) of the domestically injured was 25.1(2.0) years and a range of 3 months to 95 years. Those who sustained home injuries were younger when compared with those who sustained non-home injuries (mean age (SE), 29.0 (1.1) years), however they were older than the no-injury (mean age (SE), 23.9 (0.3) years) cohort (p = 0.56). When compared with those who sustained non-home injuries and no injury, a significantly larger proportion of those with home injuries used charcoal (p = 0.039) and liquid petroleum gasoline (LPG, p = 0.006) as cooking fuels. A greater proportion of the domestically injured owned agricultural land, and obtained a tertiary education when compared to the other groups, although these did not reach statistical significance.

**Table 1:** Comparison of demographic and socioeconomic variables between individuals with home injury, non- home injury, and the non-injured population (N = 8065*)^†^.

	Home Injury (n%)	Non- Home Injury (n%)	No Injury (n%)	p-value^a^
**Age (n = 8014)**				
**Mean (SE)**	25.1 (2.0)	29.0 (1.1)	23.9 (0.3)	0.56
**0-5years**	22 (13.9%)	7 (2.5%)	1026 (12.3%)	0.0057
**6-14years**	44 (33.1%)	38 (13.3%)	1621 (21.4%)	
**15-29years**	38 (21.7%)	122 (44.3%)	2268 (31.3%)	
**30-44years**	21 (12.2%)	83 (22.7%)	1254 (16.9%)	
**45-64years**	14 (10.6%)	43 (10.5%)	700 (8.9%)	
**≥65years**	13 (8.6%)	23 (6.7%)	677 (9.1%)	
**Sex, n = 7995**				
**Male**	69 (39.2%)	217 (66.6%)	3560 (46.1%)	0.003**
**Female**	83 (60.8%)	97 (33.4%)	3969 (53.9%)	
**Urban Residence, n = 7953**				
**Yes**	47 (41.6%)	83 (37.7%)	2203 (39.3%)	0.750
**No**	104 (58.4%)	232 (62.3%)	5284 (60.7%)	
**Own Agricultural land, n = 7949**				
**Yes**	105 (65.9%)	216 (62.4%)	4844 (56.8%)	0.110
**No**	47 (34.1%)	99 (37.6%)	2638 (43.2%)	
Cooking fuel***, **n = 8014**				
**Wood**	137 (88.7%)	294 (89.9%)	6975 (88.8%)	0.885
**Charcoal**	33 (29.6%)	44 (17.6%)	1201 (22.0%)	0.039**
**LPG**	87 (67.8%)	128 (45.4%)	3233 (52.5%)	0.006**
**Kerosene**	19 (11.2%)	52 (19.6%)	1215 (19.6%)	0.107
**Level of Education, n = 7910**				0.282
**None**	3 (2.0%)	6 (1.8%)	148 (1.2%)	
**Primary**	30 (14.8%)	77 (20.9%)	1540 (16.9%)	
**Secondary**	51 (34.0%)	116 (35.8%)	2788 (36.4%)	
**Tertiary**	64 (48.8%)	113 (41.1%)	2956 (45.2%)	
**Household possess cellphone, n = 7871**				
**Yes**	142 (95.7%)	298 (96.0%)	7075 (96.5%)	0.775
**No**	7 (4.3%)	14 (4.0%)	335 (3.5%)	

LPG = liquid petroleum gas;

^†^Percentages adjusted for the multi-cluster survey sampling method.

*Variables had missing data hence the total n differs for each variable. Missing data for each variable was excluded from analysis.

**p-value less than or equal to 0.05 considered statistically significant

***The different forms of cooking fuel are not mutually exclusive.

^a^Pearson Chi-Squared test or Adjusted Wald used as appropriate.

### Injury characteristics

The top two activities resulting in home injury were leisure or playing (51.0%) and working around the home (22.6%) (Table 2). Other activities (n = 34) at the time of injury are household chores (32.4%) and cooking (17.6%). The commonest mechanisms of home injury were falls (37.9%) followed by contact with sharp objects (28.1%). In those who sustained home injuries, lacerations were the most frequently cited injury type (59.2%). A greater proportion of individuals experiencing home injuries (14.6%) reported the type of injury being a burn as compared with individuals experiencing non-home injuries (4.5%; p = <0.001). The majority of home injuries were to the lower and upper extremities (49% and 33.8%, respectively).

**Table 2:** Characteristics of home injuries in Cameroon.

Injury Characteristics	Home Injury n (%)	Non-Home Injury n (%)	p-value
**Activity at the Time of Injury**	**N = 155**	**N = 335**	**<0.001** *
Leisure/Play	79 (51.0%)	47 (14.0%)	
Work	35 (22.6%)	130 (38.8%)	
Sport	1 (0.6%)	11(3.3%)	
Travel/Transit	0 (0.0%)	138 (41.2%)	
Other	34 (21.9%)	8 (2.4%)	
Unknown	6 (3.9%)	1 (0.3%)	
**Injury Mechanism**	**N = 153**	**N = 331**	**<0.001** *
Fall	58 (37.9%)	62 (18.7%)	
Blade/knife cut and other sharp injuries	43 (28.1%)	80 (24.2%)	
Burn from flame and scald from hot liquids	21 (13.7%)	7 (2.1%)	
Blunt force	17 (11.1%)	43 (13.0%)	
Road traffic injury	2 (1.3%)	131 (39.6%)	
Other	12 (7.8%)	8 (2.4%)	
**Injury Type** **	**N = 157**	**N = 336**	
Cut/bite/wound (laceration)	93 (59.2%)	206 (61.3%)	0.661
Burn	23 (14.6%)	15 (4.5%)	<0.001*
Sprain/strain	15 (9.6%)	54 (16.1%)	0.052
Bruise or scrape	13 (8.3%)	54 (16.1%)	0.019*
Pain (not otherwise specified)	12 (7.6%)	26 (7.7%)	0.971
Broken bone	11 (7.0%)	34 (10.1%)	0.264
Dislocation	4 (2.6%)	18 (5.4%)	0.241
Internal organ injury	2 (1.3%)	1 (0.3%)	0.239
Concussion/Brain injury	1 (0.6%)	9 (2.7%)	0.181
Other	3 (1.9%)	7 (2.1%)	1.000
**Anatomical Location of Injury** **	**N = 157**	**N = 336**	
Lower extremities	77 (49.0%)	198 (58.9%)	0.040*
Upper extremities	53 (33.8%)	103 (30.7%)	0.490
Head	21 (13.4%)	30 (8.9%)	0.131
Face	18 (11.5%)	26 (7.7%)	0.176
Abdomen	6 (3.8%)	2 (0.6%)	0.015*
Upper back	4 (2.5%)	2 (0.6%)	0.085
Lower back	4 (2.5%)	6 (1.8%)	0.733
Neck	3 (1.9%)	7 (2.1%)	1.000
Chest	3 (1.9%)	11 (3.3%)	0.564
Pelvis	3 (1.9%)	8 (2.4%)	1.000
Genitals	1 (0.6%)	0 (0.0%)	0.318
Unknown	1 (0.6%)	7 (2.1%)	0.446

*p-value less than or equal to 0.05 considered statistically significant; Pearson Chi squared or Fisher’s exact tests

**Injury types and Injured body regions not mutually exclusive

### Care-seeking and barriers to care

Overall, whereas 75.0% of those with non-home injuries sought care from a formal or informal care provider, 62.4% of those with home injuries sought care from some provider (p = 0.004). As expected, home injuries had a higher proportion of first responders providing help to the injured being family members (61.0%) compared to non-home injuries (21.1%) (p = <0.001). Those who sustained home injuries were more likely to seek treatment within the family/home (36.3% versus 24.0%, p = 0.008) or at church (2.6% versus 0%, p = 0.010). Conversely, those with non-home injuries were more likely to seek care from a traditional healer/bonesetter (14.0% versus 7.6%, p = 0.043). (Table 3).

**Table 3:** Treatment types sought after home injuries in Cameroon.

	Home Injuries [n = 157]	Non-Home Injuries [n = 336]	p-value
Care from some provider (formal/informal)	98 (62.4%)	252 (75.0%)	0.004*
Doctor/Nurse/Hospital/Clinic (formal care)	91 (58.0%)	227 (67.6%)	0.038*
Family/Home	57 (36.3%)	83 (24.7%)	0.008*
Traditional healer/Bonesetter (informal care)	12 (7.6%)	48 (14.0%)	0.043*
No treatment sought	8 (5.1%)	14 (4.2%)	0.644
Church	4 (2.6%)	0 (0.0%)	0.010*
Friend/Acquaintance	2 (1.3%)	5 (1.5%)	1.000
Other	3 (1.9%)	5 (1.5%)	0.714
Unknown	5 (3.2%)	11 (3.3%)	1.000

^†^Treatment types not mutually exclusive

* p-value less than or equal to 0.05 considered statistically significant, Pearson’s Chi-squared or Fisher’s exact tests

Similar to the non-home injuries, those with home injuries thought that the biggest problem with formal care use is that it is too expensive (27.0% versus 29.3%, p = 0.6). Amongst those who did not seek formal care first, a higher proportion of those with home injuries thought formal care was too expensive compared with non-home injuries victims (7.6% versus 3.9%, p = 0.075) or simply preferred not to seek formal care first (16.6% versus 12.8%, p = 0.262). (Table 4).

**Table 4:** Barriers to formal care utilization amongst home injured in Cameroon.

	Home Injury	Non-Home Injury	p-value*
Respondent’s opinion on the biggest barriers to formal care use**:	**n = 157 (%)**	**n = 333 (%)**	
Too expensive	46 (29.3%)	90 (27.0%)	0.600
Rude or inattentive staff	38 (24.2%)	84 (25.2%)	0.807
Inaccessible	12 (7.6%)	18 (5.4%)	0.335
Traditional medicine preferred	4 (2.6%)	5 (1.5%)	0.477
Treatment ineffective	3 (1.9%)	5 (1.5%)	0.715
Faith healing preferred	0 (0.0%)	3 (0.9%)	0.555
None	33 (21.0%)	77 (23.1%)	0.602
Other	39 (24.8%)	75 (22.5%)	0.571
Reasons for not seeking formal care first:	**n = 157 (%)**	**n = 336 (%)**	
Injury not thought to be serious	30 (19.1%)	64 (19.1%)	0.987
Patient preferences	26 (16.6%)	43 (12.8%)	0.262
Too expensive	12 (7.6%)	13 (3.9%)	0.075
Patient died before a medical center was reached	1 (0.6%)	2 (0.6%)	1.000
No access to formal health services/too far away	2 (1.3%)	9 (2.7%)	0.515
Unknown/unsure	1 (0.6%)	2 (0.6%)	1.000
Other	3 (1.9%)	8 (2.4%)	1.000

*p-value less than or equal to 0.05 considered statistically significant, Pearson’s Chi-squared test or Fisher’s exact tests

**Multiple responses allowed

### Outcomes

**Fig 1:**
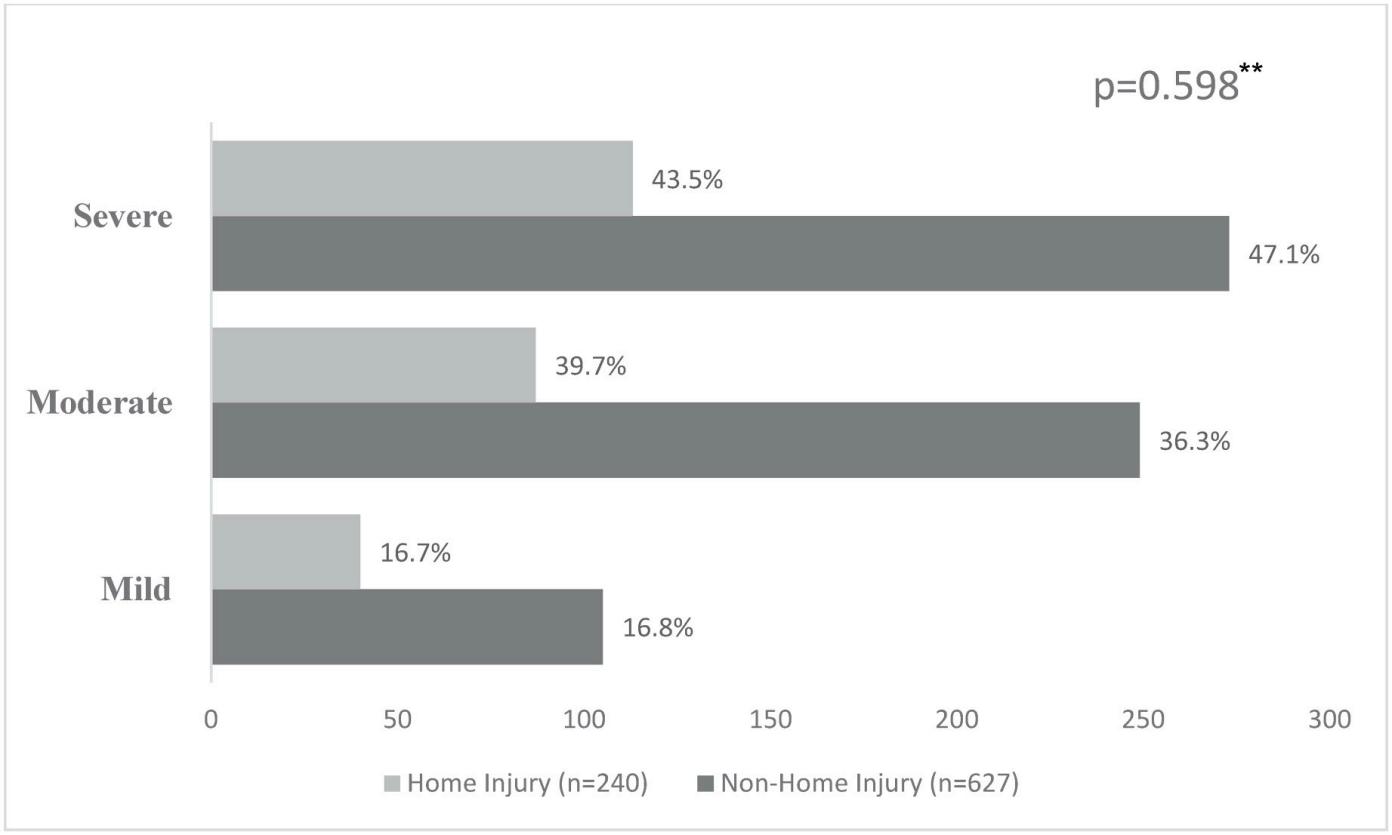
Disabilities* severity reported by home injury vs non-home injury. *Disabilities include any difficulty speaking or communicating; dressing, eating, going to the bathroom; leaving the home, shopping, traveling; engaging with friends/family; going to school; seeing or hearing; standing or walking; picking things up or using their hands; weakness, shortness of breath, fatigue; understanding or remembering things; depression or shame; Individuals could report multiple disabilities **Pearson’s Chi square.

**Fig 2:**
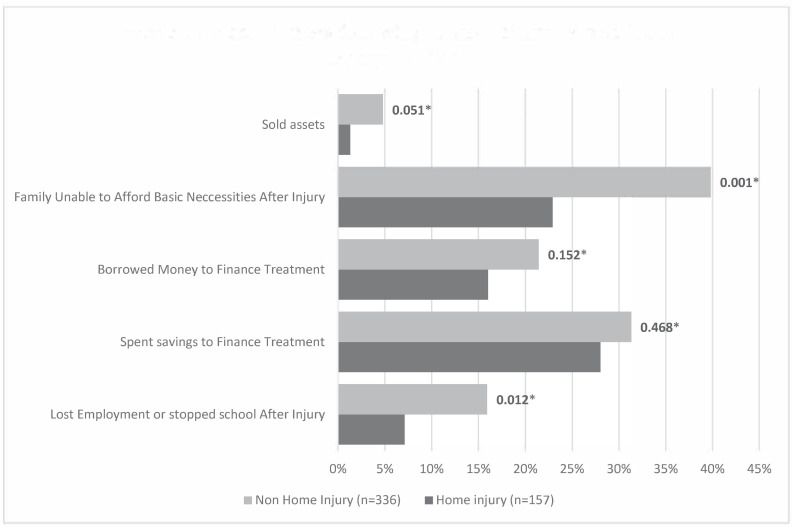
Economic consequences following home injuries in Southwest Region Cameroon. *pvalue: Pearson’s Chi squared test.

Of the 157 home injury events, 103 (65.6%) reported at least a form of disability with the top two reported disabilities being difficulties with standing/walking by 36.4% and activities of daily living (dressing, eating or going to the bathroom) by 33.8%. A total of 240 disabilities were reported with 47.1% being considered as severe (Fig 1). Interestingly, amongst those with non-home injuries, a slightly lower percentage of disabilities (43.5%) were considered by injury victims to be severe. Also, 7.1% of home injury victims completely lost their jobs or stopped going to school as a result of disabilities they sustained from their injuries (p = 0.012) while 22.9% of households were unable to afford basic necessities (food, rent) after injury (p = 0.001) (Fig 2).

The median number of disability days reported was 14 (IQR 3–30) days and the median cost of treating home injuries was 5000 (IQR 1000–15000) Central African francs (CFA). In as much as 40.4% of home injury cases, injury victims were unable to independently carry out their daily activities (p = 0.000), hence a family member had to shift their usual activities in order to care for the victim, resulting in that family member being absent from work for a median of seven days (IQR 6–18 days).

## Discussion

Through our community-based study, which provides a rare opportunity to understand home injuries at the population-level, we found that the incidence of home injury in the Southwest Region of Cameroon was 19.6 per 1000 person-years, comprising about a third of the overall burden of injury in this region. Though those who suffered home injuries reported experiencing severe disabilities more often than those who were injured outside of the home, they were less likely to seek care (formal or informal). When they did seek care, they were more likely to seek care within their family/home or their church than those injured outside the home. Interestingly, compared with those who experienced non-home injuries, those with home injuries may have been of a higher socioeconomic status (SES) based on their use of LPG [30, 31]. Most home injuries were lacerations or burns to the extremities and occurred because of falls during leisure/play or while working around the home. Finally, home injuries had significant consequences: a median disability of two weeks for the injured, with one-fifth of families facing difficulties affording basic needs.

The incidence of home injury we found was similar to that obtained by a study conducted in a semi-urban community in India (17 per 1000 person-years) [32] but much lower than that obtained from a study done in the rural area of Punjab, Northwestern India (106 per 1000 person-years) [33]. The study conducted in the rural area of India might have found a higher incidence of home injury because it seems to have employed a broader definition of home injury, based on the authors’ finding that a little more than 70% of home injuries were trivial or minor. Also, the study in rural India used a descriptive prospective epidemiological design which is less prone to recall bias than the retrospective design used in our study. Females were more affected by home injuries in our study similar to those obtained in studies done in several populations in India [32–35]. We also identified similar age groups (15 to 45years) most affected by home injuries as studies in India [33, 35]. However, our findings were different from those in a study done in Bangladesh [36], which found that males suffered more home injuries, and from other studies done in India [37, 38], which found that children under five years and adults over 65 years were the most affected age groups. The authors of the study in Bangladesh note that their finding is surprising, given that females in Bangladesh likely spend more time around the home, predisposing them to home injury. Females are also more likely to be involved in household chores and cooking which may explain their higher predisposition to home injuries. Younger and older individuals in the Indian studies might be more affected by home injury possibly because these groups spend more time at home, and also are more likely to suffer from falls. Specific interventions such as kitchen safety education can thus be directed to these groups to reduce the burden of home injuries.

Studies conducted in other countries have also found that falls are the main mechanism of home injuries [32–34, 37, 39]. We speculate that falls were common in our study because of the stony and hilly attributes of most home premises, which serve as playgrounds and work surfaces, and because of the poor floor designs (such as uneven, rough, and uncemented floors) that we observed in many participants’ homes. Further, we observed that several of the participants’ homes were poorly illuminated and were in areas where the supply of electricity is unreliable. As such, implementing preventive measures such as proper home design (including proper floors and incorporation of windows and bright lighting) may help reduce the rate of falls and therefore home injuries. Studies done in India [32–34, 37], Ghana [40], and Bangladesh [36] had similar findings and recommendations. A randomized controlled trial conducted in New Zealand amongst low-income individuals suggested that low-cost home modifications can be a means to reduce injury in the general population [39].

Victims of home injury were less likely to seek care from some care provider—despite classifying almost half of all disabilities they self-reported as severe. Although about a third of home injury victims reported the biggest barriers to formal care use as cost, the most prevalent reason they gave for not seeking formal care first was that they considered their injury not to be serious enough. Also, as reported earlier, home injury victims may have been of a higher SES due to their use of LPG which has been found to correlate with a higher SES [30, 31] than non-home injury victims, implying that, even though perceived cost of treatment was considered a substantial barrier to formal care, perception of injury severity at the time of injury might have had a bigger influence on the use of formal care. Furthermore, participants’ perception of injury as “not serious” maybe relative to expense of evaluation and treatment. Our study’s findings regarding the care-seeking behaviors of those injured domestically were similar to those reported in studies done in India [38], Bangladesh [36], and Hong Kong [41]. Subsidization of the cost of formal care treatment and implementation of a national insurance policy by the government may encourage the use of formal care facilities by the population. Our study shows that the direct economic consequences of home injury in the Southwest Region of Cameroon are serious, making salient the need for formal care subsidization and population sensitization on the impact of home injuries. Moreover, a study done in Tamil Nadu, extreme south of India found that the mean number of days of work lost for caretakers was 1.65 [42]; the median in our study was seven days. Up to 75% of home injury victims in India used formal care services, had a quicker recovery, and a lower number of post-injury disability days; in our study, only 58% of home injury victims used formal care. It is possible that access to formal care may play a role in mitigating the disability and caretaker task-shifting consequences of injury. Reducing the burden of home injuries in Cameroon may require preventing these injuries from happening in the first place, while also improving access to formal care services. Since the majority of home injury victims in our study had family members as first responders to their injury, providing appropriate first aid education/training to families during health campaigns may be an important measure in reducing disability severity, thus economic burden.

### Limitations

This study is, to the best of our knowledge, the first one at the population level that sought to understand the epidemiology and outcomes of home injury as well as care-seeking behaviors after home injury in Cameroon. However, the methods we used have some inherent limitations. In particular, we administered the survey to a single household representative who was responsible for remembering and reporting the injury events of all household members for one year. It is possible that representatives did not accurately report all injury events because, for instance, they might have been unable to recall all injury events and the relevant details, especially for minor injury events. Finally, our estimates of disability severity were necessarily based on respondents’ perception, as injuries occurred in the past, which is subject to recall bias.

## Conclusions

Home injuries comprise a significant proportion of all injuries in the Southwest Region of Cameroon. Despite being associated with increased disability compared to injuries outside of the home, as well as significant economic consequences, people who sustained home injuries are less likely to seek care. Improved safety and design of homes may improve injury prevention in this setting. To curtail the burden of disabilities and economic consequences of home injuries, efforts should be made to improve financial access to formal care services.

## Supporting information

S1 Appendix Community based household survey questionnaire. (PDF)

S1 Dataset The person-level data set used for Table 1 socio-demographic results. (DTA)

S2 Dataset The injury data set used for injury characteristics and all other presented results. (DTA)
