# Supplementary material for: Too serious to ignore: The epidemiologic and economic burden of home injuries in the Southwest Region of Cameroon—A community-based study
Source: PLoS One. 2022 Sep 22;17(9):e0274686. doi: 10.1371/journal.pone.0274686 (PMC9498948; doi:10.1371/journal.pone.0274686)
Supplement: S1 Appendix — (PDF) [file pone.0274686.s001.pdf]

Appendix. Community-Based Household Survey Questionnaire

**STUDY INFORMATION**	**Health District:**	**Health Area:**	**Interviewer Code:**	**Household Order:**	**Household #:**

**CONSENT STATUS**	☐ **Yes**<br>**Family Representative:** ___________________<br>**Family Name:**___________________________	☐ **No**<br>***If consent status switched during the survey was permission given to use previously collected data***? *Yes / No*	☐ **Not Eligible**<br>*(Nobody >= age 18, not at home after multiple attempts, or cannot understand consent)*

SECTION 1.1 Socioeconomics

**1.1.1**	**Is the household…**	☐ Rural/ More like a village? ☐ Urban/More like a city?
**1.1.2**	**Does any member of the household own a cellphone?**	☐ Yes ☐ No
**1.1.3**	**Does the household…**	☐ Own the home? ☐ Rent the home? ☐ Live for free?
**1.1.4**	**Does the household own agricultural land?**	☐ Yes ☐ No
**1.1.5**	**What types of cooking fuel are used in the household?** *(select all that apply)*	☐ Wood ☐ Charcoal ☐ Liquid Petroleum Gas (LPG) ☐ Kerosene ☐ Other
**1.1.6a-b**	a. **What is the highest education level achieved by any member of the household?**	☐ No formal education ☐ Primary School ☐ Secondary/High School ☐ Tertiary/College ☐ Unknown ☐ **Other**: **b.**_____________

SECTION 1.2 Household Members and Injury *(*Household= all persons who normally sleep under the same roof and share the same kitchen and including any such person who died in the last year)*

Starting with yourself, list the age and gender of each person living in your household* If anybody in the household died in the last year what was their age and gender?

**Subject #**	**a.Age** (years)	**b. Sex** (M/F)	**c. If deceased, date of death? *** (DD/MM/YYYY)	**d. Were any of these people injured in the past year in a way that limited normal activities for at least one day or which needed treatment? ****	**e. On how many separate occasions was each person injured in the past year?** (# separate occasions)	**f. Does anybody in the household CURRENTLY have any of the following problems?** *(write all applicable codes on the right for each person, IF THE **PROBLEM IS DUE TO INJURY** WITHIN 1 YEAR, **STAR**)*	
1 (Respondent)							
2							**1.Breast changes like lumps, cancer, skin or nipple changes or discharge**
3							
4							**2. Mass/swelling that changes size/sometimes disappears (HERNIA)**
5							
6							**3. Other mass, lump or growth**
7							
8							**4. Wound/Burn/scald**
9							
10							**5. Deformity**
11							
12							**6. Bleeding from rectum**
13							
14							**7. New blindness or lost most of their vision**
15							
16							**8. Abdominal distension OR severe/recurrent pain**
17							
18							**9. Bleeding/sickness within 6 weeks of giving birth**
19							
20							**10. Can't pass stool/urine or eat**
**Study #s:**	***If deceased, fill out Section 1.3, Cause of Death**<br>****For each separate occasion for each injured person, fill out one Section 2 form. The TOTAL Occasions should equal the number of Section 2 forms needed***				**TOTAL** _______________ **Occasions ****	**TOTAL PROBLEMS** __________ *******	******Fill out one Section 3 form for each separate problem- EXCLUDING those due to injury < 1 year ago (STARRED)***

Section 1.3 CAUSE OF DEATH How many household members died in the past 1 year? _________

1.3.1a-d	*WRITE THE SUBJECT # OF EACH DECEASED FAMILY MEMBER IN THE GREY LEFT COLUMN AND FILL 3.2.1A-D*			
If death was due to an injury for which you will fill out a Section 2 select first option for 1.3.1a. You do not need to answer 3.2.1b-d for this person.				
**Write the Subject # of all household members who DIED in the past year**	**a. Did any of the following problems occur the WEEK before death or CAUSE the death?** *(select all that apply)*	**b. If any care was sought for this problem, where?** *(Circle all that apply)*	**c. Did they have an operation for the problem?**	**d. If not, what was the reason for NOT SEEING A DOCTOR or for NOT HAVING AN OPERATION?**
__________	☐ Wound/Injury in the past 1 year<br>☐ Wound (other)<br>☐ Mass, growth or swelling<br>☐ Deformity (they were born with)<br>☐ Deformity (they developed)<br>☐ Abdominal distention or pain<br>☐ Unable to pass stool/urine, or vomits all food<br>☐ Bleeding/sickness within 6 weeks of childbirth<br>☐ Abnormal appearing newborn<br>☐ None ☐ Unknown	***formal / traditional / home / other / none / NA***	***yes / no / unknown***	☐ No need<br>☐ No money<br>☐ No transportation/ Too far<br>☐ Facility, Personnel or Equipment not available<br>☐ No time<br>☐ Preference<br>☐ Unknown<br>☐ **Other: ___________________**
__________	☐ Wound/Injury in the past 1 year<br>☐ Wound (other)<br>☐ Mass, growth or swelling<br>☐ Deformity (they were born with)<br>☐ Deformity (they developed)<br>☐ Abdominal distention or pain<br>☐ Unable to pass stool/urine, or vomits all food<br>☐ Bleeding/sickness within 6 weeks of childbirth<br>☐ Abnormal appearing newborn<br>☐ None ☐ Unknown	***formal / traditional / home / other / none / NA***	***yes / no / unknown***	☐ No need<br>☐ No money<br>☐ No transportation/ Too far<br>☐ Facility, Personnel or Equipment not available<br>☐ No time<br>☐ Preference<br>☐ Unknown<br>☐ **Other: ___________________**
__________	☐ Wound/Injury in the past 1 year<br>☐ Wound (other)<br>☐ Mass, growth or swelling<br>☐ Deformity (they were born with)<br>☐ Deformity (they developed)<br>☐ Abdominal distention or pain<br>☐ Unable to pass stool/urine, or vomits all food<br>☐ Bleeding/sickness within 6 weeks of childbirth<br>☐ Abnormal appearing newborn<br>☐ None ☐ Unknown	***formal / traditional / home / other / none / NA***	***yes / no / unknown***	☐ No need<br>☐ No money<br>☐ No transportation/ Too far<br>☐ Facility, Personnel or Equipment not available<br>☐ No time<br>☐ Preference<br>☐ Unknown<br>☐ **Other: ___________________**
__________	☐ Wound/Injury in the past 1 year<br>☐ Wound (other)<br>☐ Mass, growth or swelling<br>☐ Deformity (they were born with)<br>☐ Deformity (they developed)<br>☐ Abdominal distention or pain<br>☐ Unable to pass stool/urine, or vomits all food<br>☐ Bleeding/sickness within 6 weeks of childbirth<br>☐ Abnormal appearing newborn<br>☐ None ☐ Unknown	***formal / traditional / home / other / none / NA***	***yes / no / unknown***	☐ No need<br>☐ No money<br>☐ No transportation/ Too far<br>☐ Facility, Personnel or Equipment not available<br>☐ No time<br>☐ Preference<br>☐ Unknown<br>☐ **Other: ___________________**
__________	☐ Wound/Injury in the past 1 year<br>☐ Wound (other)<br>☐ Mass, growth or swelling<br>☐ Deformity (they were born with)<br>☐ Deformity (they developed)<br>☐ Abdominal distention or pain<br>☐ Unable to pass stool/urine, or vomits all food<br>☐ Bleeding/sickness within 6 weeks of childbirth<br>☐ Abnormal appearing newborn<br>☐ None ☐ Unknown	***formal / traditional / home / other / none / NA***	***yes / no / unknown***	☐ No need<br>☐ No money<br>☐ No transportation/ Too far<br>☐ Facility, Personnel or Equipment not available<br>☐ No time<br>☐ Preference<br>☐ Unknown<br>☐ **Other: ___________________**

SECTION 2: INJURY OCCASION

2.1 Subject #____________ Separate Occasion # _____*(1st occasion=1, 2nd occasion=2, etc.)* **(NAME_________________)**

2.1.1 **Date of Injury** *(approximate date if cannot remember)* ________________ DD/MM/YYYY

2.2 Pre-Injury Activities

2.2.1 **How many years of school has the injured person completed?** ______________years

2.2.2a-c

a. In the 30 days prior to injury what job or activity did the injured person spend most of their time doing?

____

____

When entering data, select occupation and industry based on this description and using the ECONOMIC CODE SHEET.
i. OCCCUPATION #______ ii.INDUSTRY #_____ Coder_______

b. How many hours per week did they do this activity? __________________**hours/week**

c. About **how much did they earn doing this activity?** ___________**CFA per** *(circle one)* **day / week / month / year**

2.2.3 **Did the person have another major job or activity?**

☐ Yes ☐ No ☐ Unknown

If "NO" or "UNKNOWN" skip to ***Section 2.3***

2.2.4a-c

a. If yes, describe:

____

____

When entering data, select occupation and industry based on this description and using the ECONOMIC CODE SHEET.
i. OCCCUPATION #______ ii.INDUSTRY #_____ Coder ______

b. How many hours per week did they do this activity? __________________**hours/week**

c. About how much did the person earn doing this activity? ___________**CFA per** *(circle one)* **day / week / month / year**

2.3 Injury Characteristics

2.3.1a-b **a. What was the person doing when they became injured?**

☐ Work ☐ School ☐ Travel/Transit ☐ Sport ☐ Leisure/Play ☐ Unknown ☐ **Other:b.**_________

2.3.2a-b **a. Where did the injury occur?**

☐ At home	☐ Railway line	☐ Farm	☐ Sports area	☐ Unknown
☐ Someone else's home	☐ Construction site	☐ Body of water	☐ School	☐ **Other:**
☐ Street/highway	☐ Trade/Service area	☐ Open land	☐ Public area	**b.**______________

2.3.3a-b **a. What caused the injury?**

☐ **Road Traffic Injury**	☐ Firearm wound	☐ Burn from flame/heat	☐ Poisoning	☐ Unknown
☐ Fall	☐ Other sharp injury	☐ Scald from hot liquid	☐ Drowning	☐ **Other:**
☐ Blunt force (object/person)	☐ Electrocution	☐ Smoke inhalation	☐ Animal bite	**b.**______________
☐ Blade/knife cut		☐ Explosive blast		

If "Road Traffic Injury" was NOT selected, skip to ***Question 2.3.5***

2.3.4a-d

a. If Road Traffic Injury, what types(s) of transportation were involved? *(select all that apply)*

☐ Pedestrian	☐ Taxi	☐ Truck/Lorrie	☐ Bicycle
☐ Private car	☐ Mototaxi	☐ Bus	☐ Unknown
☐ Private motorcycle	☐ Minivan/minibus	☐ Train	☐ **Other**
			**b.**__________

c. If RTI, were seatbelts used? ☐ Yes ☐ No ☐ NA

d. If RTI, were helmets worn? ☐ Yes ☐ No ☐ NA

2.3.5 **Was the injury accidental or did somebody do this on purpose?**

☐ It was an accident (unintentional) ☐ Someone else did it to them on purpose ☐ They did it to themselves on purpose ☐ Unknown/Unsure

2.3.6 **Point to and tell me which part(s) of the body were injured…** *(select all that apply)*

☐ Head	☐ Back at/above 12th rib	☐ Abdomen	☐ Upper extremities
☐ Face	☐ Back below 12th rib	☐ Pelvis	☐ Lower extremities
☐ Neck	☐ Chest and torso	☐ Genitals	☐ Unknown

2.3.7a-b **a. How was the person hurt?** (*Select all that apply)* ☐ Poisoning

☐ Broken bone	☐ Cut/bite/wound	☐ Concussion/Brain injury	☐ Unknown/unsure
☐ Sprain/strain	☐ Bruise or scrape	☐ Internal organ injury	☐ Other:
☐ Dislocation	☐ Burn	☐ Pain (not otherwise specified)	**b.**______________________

2.3.8 **On the day of the injury, did the injured person?** *(Select all that apply)* ☐ None

☐ Die ☐ Stop breathing ☐ Lose Consciousness ☐ Forget the injury ☐ Act confused ☐ Unknown

2.4 Injury Treatment

2.4.1a-b	**a. What type of person first provided help to the injured person?**<br>□ Family member □ Police officer □ Unknown<br>□ Friend/ acquaintance □ Taxi/Mototaxi driver □ **Other:**<br>□ Bystander/stranger □ No help was provided **b.**________________
2.4.2a-b	**a. How was the injury treated?** (*mark all that apply. Indicate the numeric order if known (1= first care type sought, 2= 2nd type sought…)*)<br>____No treatment sought ____Friend/acquaintance ____Traditional healer/ bonesetter ____ Unknown<br>____Family/ Home treatment ____Church ____Doctor/nurse/hospital/clinic ____ **Other b.**____________
2.4.3	**Approximately how much did all treatment cost (CFA)?** ____________CFA<br>*(Including outpatient costs, inpatient costs, laboratory fees, imaging fees, medicines, dressings, supplies, procedure fees, transport costs, and food costs above what the family would normally pay)*<br>If HR gives itemized cost listing note below:<br>____________CFA for<br>____________CFA for<br>____________CFA for
	If formal care WAS sought FIRST skip to ***Question 2.4.5***
2.4.4a-b	**a. If formal medical care (doctor /nurse…) was not sought FIRST which best describes why?**<br>□ Injury not serious □ Patient preference<br>□ Patient died before medical center was reached □ Unknown/Unsure<br>□ Too expensive □ **Other:b.**________________<br>□ No access to formal health services/ too far away ________________
	If formal care WAS NOT sought AT ALL skip to ***Section 2.5.***<br>*When survey is completed, inform Qualitative RA household meets criteria for* ***injured non-presenters****.*
2.4.5	**How many nights did the person stay in the hospital?** ________________nights
2.4.6	**Did the patient receive an operation as part of their treatment?** □ Yes □ No □ Unknown
2.4.7a-b	**a. Why did the injured person leave the hospital?**<br>□ They died □ They were evaluated but never admitted<br>□ They were sent home by staff (discharged) □ Unknown/ Unsure<br>□ They went home against medical recommendation □ **Other: b.** ________________
2.4.8	**Overall, how satisfied were you with the medical care your family member received?**<br>□ Satisfied<br>□ Neutral<br>□ Dissatisfied
2.4.9	**Did your family experience any of the following while seeking formal care?** *(select all that apply)*<br>□ Long wait times<br>□ Supplies or medicines unavailable<br>□ Specialist unavailable<br>□ Unable to pay for supplies, medicines, or care<br>□ Rude or disrespectful treatment by staff<br>□ Non- functioning equipment<br>□ Poorly kept facilities

2.5 Mortality

	If the injured person is STILL ALIVE (as indicated in Section 1), skip to ***Section 2.6***
2.5.1	**How long after injury did the person die?**<br>□ Immediately □ >24 hours later<br>□ <6 hours later □ Unknown<br>□ 6- 24hours later
2.5.2	**Would the person have died if they had not been injured?**<br>□ Yes □ No □ Unknown/Unsure

2.6 Disability

	a. Following injury did they have any NEW or WORSE difficulty with… *(Select best option for each)*				b. If yes, was the disability? *(Circle)*	c. Is this still a problem now? *(Circle)*
2.6.1a-c	**i. Speaking or communicating?**	___No	___Yes	___ Unknown	mild / moderate/ severe	yes / no / unknown
	**ii. Dressing, eating, going to the bathroom?**	___No	___Yes	___ Unknown	mild / moderate/ severe	yes / no / unknown
	**iii. Leaving the home, shopping, traveling?**	___No	___Yes	___ Unknown	mild / moderate/ severe	yes / no / unknown
	**iv. Engaging with friends/ family?**	___No	___Yes	___ Unknown	mild / moderate/ severe	yes / no / unknown
	**v. Going to school?**	___No	___Yes	___ Unknown	mild / moderate/ severe	yes / no / unknown
	**vi. Seeing or hearing?**	___No	___Yes	___ Unknown	mild / moderate/ severe	yes / no / unknown
	**vii. Standing or walking?**	___No	___Yes	___ Unknown	mild / moderate/ severe	yes / no / unknown
	**viii. Picking things up or using their hands?**	___No	___Yes	___ Unknown	mild / moderate/ severe	yes / no / unknown
	**ix. Weakness, shortness of breath, fatigue?**	___No	___Yes	___ Unknown	mild / moderate/ severe	yes / no / unknown
	**x. Understanding or remembering things?**	___No	___Yes	___ Unknown	mild / moderate/ severe	yes / no / unknown
	**xi. Depression or shame?**	___No	___Yes	___ Unknown	mild / moderate/ severe	yes / no / unknown

2.6.2	**Did the injured person lose their job or completely stop going to school because they had been injured?**<br>□ Yes □ No □ Unknown □ NA
2.6.3a-d	**a. Has the patient been readmitted to the hospital or needed additional treatment for problems related to their injury?**

(NOT including scheduled follow-up visits)

☐ **Yes** ☐ No ☐ Unknown

es, how many..
________ re-admissions?
________ consultations/ visits?
________ operations?

2.6.4 **Will the injured person likely need further medical care in the future for problems related to their injury?**
☐ Yes ☐ No ☐ Unknown

2.7 Economic Impact

2.7. 1 **After being hurt, was there at least one day the injured person was not able to perform their main job/ activity by themselves?**
☐ Yes ☐ No ☐ Unknown

If "NO" or "UNKNOWN" skip to ***Question 2.7.3***

2.7.2 a-c
a. If yes, for how many days? __________days
b. How much of the job/activity was completed by others? ☐ All of it ☐ Most of it ☐ Some of it ☐ Unknown/NA
c. Who performed or helped them perform the job/activity? ☐ Family ☐ Non-family ☐ Nobody ☐ Unknown/NA

If the injured person DID NOT have a second major job or activity (Question 2.2.3), skip to ***Question 2.7.5***

2.7.3 **After being hurt, was there at least one day the injured person could not perform their secondary job/activity by themselves?**
☐ Yes ☐ No ☐ Unknown

If "NO" or "UNKNOWN" skip to ***Question 2.7.5***

2.7.4a-c
a. If yes, for how many days? ___________days
b. How much of the job/activity was completed by others? ☐ All of it ☐ Most of it ☐ Some of it ☐ Unknown/NA
c. Who performed or helped them perform the job/activity? ☐ Family ☐ Non-family ☐ Nobody ☐ Unknown/NA

2.7.5a-b
a. After being hurt, was there even one day another family member had to shift their usual activities to care for the injured person (CARETAKER)?
☐ **Yes** ☐ No ☐ Unknown
b. If yes, for how many days? ______days

If "NO" or "UNKNOWN" skip to ***Question 2.7.9***

2.7.6a-c
a. In the 30 days prior to the person's injury what job/activity did the CARETAKERS spend most of their time doing________________________________?

When entering data, select occupation and industry based on this description and using the ECONOMIC CODE SHEET.
i. OCCCUPATION #______ ii. INDUSTRY #_____ Coder_______

b. How many hours per week did they do this activity? _________________**hours/week**
c. About how much did the person earn doing this activity? ___________**CFA per** *(circle one)* **day / week / month / year**

2.7.7 **In the 30 days prior to the injury, did the CARETAKER have another major job/activity?**
☐ Yes ☐ No ☐ Unknown

If "NO" or "UNKNOWN" skip to ***Question 2.7.9***

2.7.8a-c
a. If yes, describe:________________________________
____

When entering data, select occupation and industry based on this description and using the ECONOMIC CODE SHEET.
i. OCCCUPATION #______ ii. INDUSTRY #_____ Coder_______

b. How many hours per week did they do this activity? _________________**hours/week**
c. About how much did the person earn doing this activity? ___________**CFA per** *(circle one)* **day / week / month / year**

2.7.9 **Following the injury, did it become more difficult for the family to afford expenses such as food and rent?**
☐ Yes ☐ No ☐ Unknown

2.7.10a-d
a. Following the injury did the family do any of the following? *(select all which apply)*
☐ Sell assets (livestock, vehicles, property etc.) — b. **If so, how much was made from the sale?** _____________CFA
☐ Spend saved money — c. **If so, how much was spent?** _____________CFA
☐ Borrow money — d. **If so, how much was borrowed?** _____________CFA
☐ None
☐ Unknown

2.7.11 **How long do you expect the injured person's new limitations to last?**
☐ They recovered ☐ ________ *(circle one)* **day / week / month / year** ☐ Forever ☐ Unknown

Section 4: Opinions of Formal Care and Health Knowledge Assessment

4.1	**Where would your family most likely go for medical care after injury?** ___________________________
4.2	**In your opinion, what is the biggest problem with formal medical care services in Southwest Cameroon?**<br>□ Too expensive<br>□ Too far away/difficult to reach<br>☐ The treatments don't work<br>□ Rude behavior by staff<br>□ Traditional medicine is preferred<br>☐ Faith treatment is preferred<br>☐ None<br>□ **Other: ii.**_______________________________________
4.3 a-b	**a. Do you know how to get to the nearest health facility?** □ Yes □ No<br>**b. If "Yes" what type of transportation would you most likely use to travel there?** __________________________<br>**c. Using this method, approximately how long do you think it would it take to travel there?** _______hours
4.4	**Which of the following conditions do you believe can be treated or reversed with surgery?** □ None<br>*(select all that apply)*<br>□ Some types of blindness □ Some types of cancer □ Injury □ Burns □ Deformities

*If the respondent is **MALE** please skip to **Section 5***

4.5 a-c	**Do you know how to perform breast self-exam?**<br>□ No, I've never heard of it □ No, I have heard of it but don't know how to do it □ Yes, I know how to do it	

*If "No…" was selected, skip to **Section 5***

	b. **How often have you done breast self-exam in the past year?**<br>□ Monthly<br>□ Several times<br>□ Once<br>□ None<br>□ I have NEVER done it	c. **What time of the month do you perform self-breast exam?**<br>□ At any time<br>□ First week after menses<br>□ Second week after menses<br>□ Menses

Section 5: LANGUAGE BARRIERS IN FORMAL HEALTHCARE

5.1a-c	**a. What is your region of origin?**<br>□ Southwest, Cameroon<br>□ **Other region in Cameroon** b. **Specify:**_________________________<br>□ **Other country** c. **Specify country:**_______________
5.2a-b	**a. Which language is spoken by the household most often at home?**<br>□ Pidgin English □ English □ French □ **Local language or other: b.**_______
5.3a-b	**a. Which other languages are spoken by household members ?** *(select all that apply)*<br>□ Pidgin English □ English □ French □ **Local language or other: b.**_______
5.4	**If you felt a doctor did not understand you, what would you most likely do?**<br>□ Find another patient or family member to help translate<br>□ Follow instructions to my best ability using what I do understand<br>□ Find a different doctor<br>□ Leave and go to a traditional healer<br>□ Leave and treat the problem at home<br>□ **Other: b.**____________________________
5.5	**Has anyone in the household ever gone to a hospital/medical center for any reason?**<br>□ Yes □ No □ Unknown

If "Yes" ask questions 5.5 – 5.9. If "No" or "Unknown" end survey

5.6a-b	**a. If yes, what languages were spoken with staff?** *(select all that apply)*<br>□ Pidgin English □ English □ French □ **Local language or other: b.**____________	
5.7a-c	**How much did the household member(s) understand:**	*(For each letter circle best answer)*
	**a. what the doctor told them about the problem?**	All of it / Most of it / Some of it / Very little / None of it / Unknown / NA
	**b. instructions for how to take medicines correctly?**	All of it / Most of it / Some of it / Very little / None of it / Unknown / NA
	**c. instructions for caring for the problem after returning home?**	All of it / Most of it / Some of it / Very little / None of it / Unknown / NA
5.8	**Did the hospital staff understand what the household member(s) said to them?** *(circle best answer)*	All of it / Most of it / Some of it / Very little / None of it / Unknown
5.9	**Has anyone in the household brought another person to the hospital/medical center to help them translate?**<br>□ Yes □ No □ Unknown	
